# An online tool for predicting ovarian responses in unselected patients using dynamic inhibin B and basal antimüllerian hormone levels

**DOI:** 10.3389/fendo.2023.1074347

**Published:** 2023-01-20

**Authors:** Congcong Ma, Huiyu Xu, Haiyan Wang, Guoshuang Feng, Yong Han, Kannan Alpadi, Rong Li, Jie Qiao

**Affiliations:** ^1^ Center for Reproductive Medicine, Department of Obstetrics and Gynecology, Peking University Third Hospital, Beijing, China; ^2^ National Clinical Research Center for Obstetrics and Gynecology (Peking University Third Hospital), Beijing, China; ^3^ Key Laboratory of Assisted Reproduction (Peking University), Ministry of Education, Beijing, China; ^4^ Beijing Key Laboratory of Reproductive Endocrinology and Assisted Reproductive Technology, Beijing, China; ^5^ Big Data Center, Beijing Children’s Hospital, Capital Medical University, National Center for Children’s Health, Beijing, China; ^6^ Hangzhou Qingguo Medical Technology Co. Ltd., Hangzhou, Zhejiang, China; ^7^ The Predict Health, Houston, TX, United States; ^8^ Beijing Advanced Innovation Center for Genomics, Beijing, China; ^9^ Peking-Tsinghua Center for Life Sciences, Peking University, Beijing, China

**Keywords:** inhibin B, antimüllerian hormone, ovarian response, controlled ovarian stimulation, predictive model

## Abstract

**Background:**

Reliable predictive models for predicting excessive and poor ovarian response in controlled ovarian stimulation (COS) is currently lacking. The dynamic (Δ) inhibin B, which refers to increment of inhibin B responding to exogenous gonadotropin, has been indicated as a potential predictor of ovarian response.

**Objective:**

To establish mathematical models to predict ovarian response at the early phase of COS using Δinhibin B and other biomarkers.

**Materials and methods:**

Prospective cohort study in a tertiary teaching hospital, including 669 cycles underwent standard gonadotropin releasing hormone (GnRH) antagonist ovarian stimulation between April 2020 and September 2020. Early Δinhibin B was defined as an increment in inhibin B from menstrual day 2 to day 6 through to the day of COS. Least Absolute Shrinkage and Selection Operator (LASSO) logistic regression with 5-fold cross-validation was applied to construct ovarian response prediction models. The area under the receiver operating characteristic curve (AUC), prevalence, sensitivity, and specificity were used for evaluating model performance.

**Results:**

Early Δinhibin B and basal antimüllerian hormone (AMH) levels were the best measures in building models for predicting ovarian hypo- or hyper-responses, with AUCs and ranges of 0.948 (0.887–0.976) and 0.904 (0.836–0.945) in the validation set, respectively. The contribution of the early Δinhibin B was 67.7% in the poor response prediction model and 56.4% in the excessive response prediction model. The basal AMH level contributed 16.0% in the poor response prediction model and 25.0% in the excessive response prediction model. An online website-based tool (http://121.43.113.123:8001/) has been developed to make these complex algorithms available in clinical practice.

**Conclusion:**

Early **Δ**inhibin B might be a novel biomarker for predicting ovarian response in IVF cycles. Limiting the two prediction models to the high and the very-low risk groups would achieve satisfactory performances and clinical significance. These novel models might help in counseling patients on their estimated ovarian response and reduce iatrogenic poor or excessive ovarian responses.

## Introduction

Ovarian response, defined as the number of oocytes retrieved during controlled ovarian stimulation (COS), is a keystone for success of *in vitro* fertilization (IVF) (1, 2). Excessive response during ovarian stimulation indicates the potential risk of ovarian hyperstimulation syndrome (OHSS), which is a serious iatrogenic complication of COS (3, 4). Predicting excessive ovarian response to early identify those hyper-responders would allow early interventions thus minimizing potential complications. Likewise, failure to respond adequately with few oocytes retrieved during COS is called poor ovarian response (POR), resulting in a high risk of cycle cancellation and a significantly diminished probability of pregnancy (1). Fortunately, there are some options, such as dose adjustment of follicle-stimulating hormone (FSH), for an identified abnormal ovarian response in the early stage of COS. Therefore, an accurate predictive system to predict excessive or poor ovarian response in the IVF cycle is critical to both optimize outcomes and reduce complications.

Several factors are widely used to help clinicians early identify ovarian response, including age, antral follicle counts (AFC), AMH, basal FSH levels, basal estradiol levels and luteinizing hormone (LH)/FSH ratio (5–14). Although evidence indicates that AMH and AFC may be superior to others in predicting ovarian responses (10, 13, 15–17), a reliable predictive model in all clients is currently lacking. The above facts promote the need for the establishment of sophisticated models using multiple factors to predict ovarian response.

Serum inhibin B has been advocated for decades as one of the potential novel biomarkers in predicting ovarian response. It is produced by the cohort of developing preantral and early antral follicles under the stimulation of FSH, and it peaks at the mid-follicular phases (18, 19). Early follicular inhibin B levels decrease during reproductive aging, leading to increased FSH concentrations (20). Although the absolute value of inhibin B at the start of COS remains controversial to reflect ovarian reserve, the dynamic (Δ) inhibin B, which refers to increment of inhibin B responding to exogenous gonadotropin, has been indicated as a better predictor of ovarian response than AFC and AMH (21, 22).

To take early measures to reduce iatrogenic POR thus reducing the unnecessary ovarian puncture, as well as to reduce the excessive ovarian response thus reducing the incidence of OHSS, we aimed to establish multivariate mathematical models to predict excessive and poor ovarian response at the early phase of COS, using early Δinhibin B (defined as an increase in the inhibin B level on menstrual day 6 from menstrual day 2) and other biomarkers.

## Materials and methods

### Subjects

This was a prospective observational cohort study involving a continuous series of women seeking IVF from April 2020 to September 2020 in Peking University Third Hospital. Patients undergoing standard GnRH antagonist ovarian stimulation were included. There were no exclusion criteria for the participant recruitment. Participants all provided written informed consent. Approval from the Human Reproductive Ethics Committee of Peking University Third Hospital was obtained (2015sz-017).

### Controlled ovarian stimulation

The COS protocol was as described (5, 6). Briefly, recombinant human FSH (rFSH) treatment was initiated on menstrual day 2. On menstrual day 6, the follicle growth was monitored by ultrasound and the dosage of rFSH was adjusted according to follicle growth and E_2_ levels. The GnRH antagonist treatment was initiated on stimulation day 5–7 when the growing follicles were 10–12 mm in diameter. When at least 2 dominant follicles (diameter ≥18 mm) were observed by ultrasound, 5000-10000 IU hCG was administered to trigger final oocyte maturation. Thirty-six hours after hCG administration, oocyte retrieval was performed. Then, embryos were transferred to the mother after culture or cryopreserved for future use.

### Endocrine assays

On menstrual days 2 and day 6 of the COS treatment cycle, intravenous blood was collected for measuring LH, progesterone, estradiol (E_2_), FSH, testosterone (TES), androstenedione (AND), AMH and inhibin B concentrations. Blood samples were collected, and the collection tubes immediately inverted five times and centrifuged (1800g, 10min) for further endocrine assessments.

Serum levels of FSH, LH, E_2_, progesterone, TES, and AND were tested using a Siemens Immulite 2000 immunoassay system (Siemens Healthcare Diagnostics, Shanghai, P. R. China). Quality control samples were supplied by Bio-RAD Laboratories (Hercules, CA, USA; Lyphochek Immunoassay Plus Control, Trilevel, catalog number 370, lot number 40370). Serum AMH and inhibin B concentrations were measured using an ultrasensitive two-site ELISA (Ansh Labs LLC; Webster, TX, USA), using quality controls supplied with the kit. For the Trilevel controls, the coefficients of variation for the assays were <6% for AMH, inhibin B, FSH and LH, and <10% for E_2_, progesterone, AND, and TES.

### Clinical outcomes

The outcome variables were POR or excessive ovarian response. POR was defined as <5 oocytes retrieved, and the excessive ovarian response was defined as >20 oocytes retrieved.

### Development of predictive models

Sixteen potential candidate variables were considered for inclusion in poor and excessive ovarian response prediction models, including age, basal hormone levels on menstrual day 2 (inhibin B, AMH, LH, FSH, E_2_, progesterone, TES and AND) and changed hormone levels on menstrual day 6 from day 2 (Δinhibin B, ΔAMH, ΔLH, ΔE_2_, Δprogesterone, ΔTES and ΔAND). Prior to modeling, we first explored the distribution of the independent variables. Because the distribution of Δinhibin B, basal AMH, and ΔE_2_ were obviously skewed, logarithmic transformation of these three variables was performed. The Bayesian Information Criterion (BIC) value was calculated through univariate logistic regression to evaluate whether the transformed data were better than the original data for constructing the prediction models.

Seventy percent of the data were selected randomly as the training set, which was used for model establishment, and the rest 30% of the data were used as the validation set, which was used for model evaluation. For variable selection, the sixteen variables were entered into the selection process. There is a strong correlation between the variables, as shown in Supplemental **Figure 1**. To minimize the potential collinearity of variables and overfitting, Least Absolute Shrinkage and Selection Operator (LASSO) regression was used to filter the variables. The optimal model was established *via* 5-fold cross-validation. The criterion for variable selection was the scaled negative log-likelihood in the training set, which considered the one with the smallest scaled negative log-likelihood as the best. The number of predictors selected for constructing the final model was also adjusted to balance both the accuracy and simplicity of the model. The main effect of each included variable reflects the relative contribution of the variable alone, and the total effect represents the relative contribution of that variable both alone and in combination with other variables.

**Figure 1:**
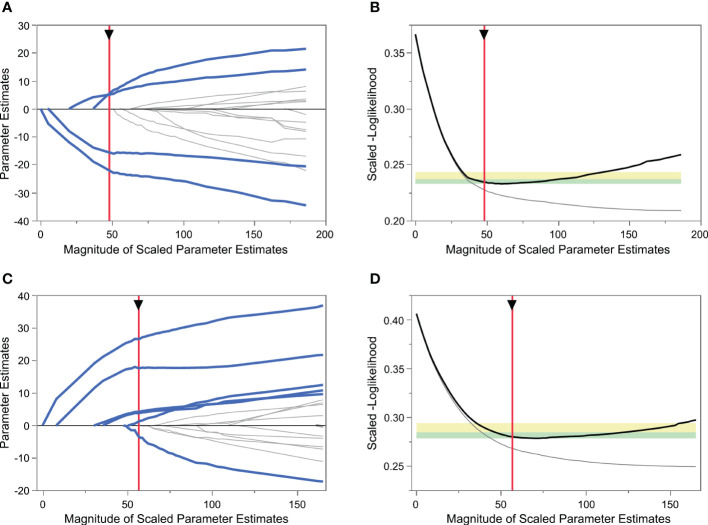
Variable selection using the least absolute shrinkage and selection operator (LASSO) logistic regression model. **(A)** LASSO model showing coefficient profiles of the candidate variables for predicting poor ovarian response (POR). Each trace represents a regression parameter, and the penalty decreases from left to right. **(B)** Tuning parameter selection of POR prediction model by 5-fold cross-validation. The vertical red axis indicates the optimal values on the basis of the minimum scaled[-log(likelihood)]. **(C)** LASSO model coefficient profiles of the candidate variables for excessive ovarian response prediction. **(D)** Tuning parameter selection of excessive ovarian response prediction model by 5-fold cross-validation.

### Evaluation of model performance

The performance of our model was assessed by calculating the area under the receiver-operator characteristic curve (AUC), sensitivity, specificity, positive predictive value (PPV) and negative predictive value (NPV). When the model was evaluated as being satisfactory, we aimed to develop an easy-to-use clinician-friendly online tool to stratify patients as having a certain risk of poor or excessive ovarian response.

### Statistical analysis

Normally distributed variables are shown as the mean and standard deviation, while variables not normally distributed are presented as the median and interquartile range (IQR). An independent samples t-test or non-parametric test was used to analyze continuous variables. All the analyses in this study were performed using SAS JMP Pro (version 14.2; SAS Institute, Cary, NC, USA), and *p* < 0.05 was considered statistically significant.

## Results

### Patient demographics

We collected 669 standard GnRH antagonist COS cycles prospectively without data selection. After excluding 27 cycles in which the numbers of oocytes retrieved were not recorded, 642 cycles were finally included for analysis. Participants had a mean age of 32.8 years (range 22-45 years) in this study. The basic characteristics of the cycle data are shown in **Table 1**. A total of 467 patients (72.7%) were normal responders and the median number of obtained oocytes was 11 (IQR 8-16). Ninety-two patients (14.3%) were diagnosed as excessive responders and the median number of obtained oocytes was 25 (IQR 22-28). The remaining 83 patients (12.9%) were diagnosed as poor responders and the median number of obtained oocytes was 2 (IQR 2-3). In the poor responder group, three patients had failed oocyte retrieval.

**Table 1:** Clinical characteristics of 642 IVF cycles of standard GnRH antagonist ovarian stimulation.

Variable		
Age (y), mean ± SD	32.77 ± 4.32	
BMI (kg/m^2^), mean ± SD	22.48 ± 3.42	
Main cause of infertility, n (%)		
Male factors	190 (29.60%)	
Tubal factor	173 (26.95%)	
PCOS	105 (16.36%)	
Uterine cavity factors	53 (8.26%)	
Endometriosis	41 (6.39%)	
Unexplained cause	80 (12.46%)	
Serum hormones	Basal levels	Δlevels
AMH (ng/mL), median (IQR)	3.00 (1.60, 5.30)	-0.51 (-1.22, -0.16)
FSH (IU/L), median (IQR)	6.30 (5.20, 7.90)	–
LH, median (IQR)	3.40 (2.40, 4.80)	-1.97 (-3.01, -1.15)
E_2_ (pmol/L), median (IQR)	152.00 (122.00, 176.00)	1126.00 (484.00, 2127.75)
Progesterone, median (IQR)	1.20 (1.00, 1.40)	-0.22 (-0.49, 0.00)
TES, median (IQR)	0.70 (0.70, 0.80)	0.00 (0.00, 0.15)
AND, median (IQR)	6.60 (5.00, 9.20)	0.89 (-0.77, 2.92)
Inhibin B, median (IQR)	88.50 (63.40, 114.23)	649.14 (325.75, 1187.69)
Peak E_2_ (pg/ml), median (IQR)	2480.00 (1577.00, 3857.00)	
Total rFSH dose (IU), median (IQR)	2325 (1725, 3150)	
Obtained oocytes, median (IQR)	11.00 (7.00, 17.00)	
Ovarian response, n (%)		
Normal ovarian response	467 (72.74%)	
Poor ovarian response	83 (12.93%)	
Excessive ovarian response	92 (14.33%)	

BMI, body mass index; PCOS, polycystic ovary syndrome; AMH, antimüllerian hormone; FSH, follicle stimulating hormone; LH, luteinizing hormone; E_2_, estradiol; TES, testosterone; AND, androstenedione; rFSH, recombinant follicle stimulating hormone. -, not available; Δ, dynamic.

### Establishment of ovarian response prediction models

After logarithmic transformation of the Δinhibin B, basal AMH and ΔE_2_ measures, the BIC of each predictor before and after transformation was decreased significantly, from 387.90 to 332.65 for Δinhibin B, from 401.31 to 351.28 for basal AMH and from 445.31 to 424.74 for ΔE_2_, indicating the advantages of data transformation.

The process of variable selection using LASSO and fivefold cross-validation is shown in **Figure 1**. When predicting POR, the normal responders and excessive responders were grouped as non-poor ovarian responders. As shown in **Figures 1A, B**, the scaled [–log(likelihood)] value in the validation set was smallest when four variables were included, thus including more variables did not induce a decrease of the scaled [–log(likelihood)]. Based on Occam’s razor principle (23), four variables were finally included in the POR prediction model (Model 1), namely, log[Δinhibin B], log[basal AMH], basal FSH, and ΔTES.

Similarly, when predicting excessive ovarian response, the normal responders and poor responders were grouped as non-excessive ovarian responders. Using the same method, six variables including log[Δinhibin B], log[basal AMH], basal FSH, ΔAND, basal AND, and basal LH were selected into the excessive ovarian response prediction model (Model 2) (**Figures 1C, D**).

The main and total effects of each variable in Model 1 predicting POR and Model 2 predicting excessive ovarian response were shown in **Supplemental Table 1**. In both models, the most important variable was log[Δinhibin B], with a main effect of 67.7% and total effect of 77.7% in Model 1, and with main effect of 56.6% and total effect of 69.2% in Model 2. Log[basal AMH] was the second important variable in both two models, with main effect of 16.0% and total effect of 25.0% in Model 1, and with main effect of 22.1% and total effect of 33.3% in Model 2. In order to find a simpler model for predicting excessive ovarian response, we tried to build another model using only these two most important predictors (Model 2s). The main and total effects of log[Δinhibin B] in Model 2s were 56.4% and 75.0%, and of log[basal AMH] 25.0% and 43.5%, respectively.

### Performances of established prediction models

**Figure 2:**
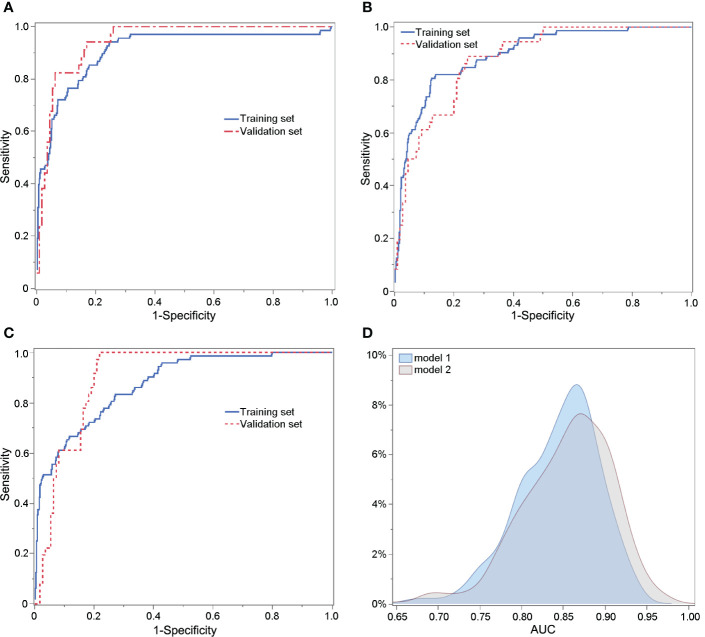
ROC curves of Model 1 predicting poor ovarian response **(A)**, Model 2 **(B)** and Model 2s **(C)** for predicting excessive ovarian response. **(D)** Comparison of the AUCs of Models 2 and 2s using 1000 × bootstrap sampling in the validation set.

The performances of Models 1, 2, and 2s are shown in **Supplemental table 2**. The AUC values of Model 1, Model 2 and Model 2s in the training set were 0.910, 0.896 and 0.875; in the validation set they were 0.948, 0.882 and 0.904, respectively. The sensitivity and specificity of Model 1 were 0.456 and 0.978, of Model 2 0.431 and 0.980 and of Model 2s 0.389 and 0.984, respectively. The PPV and NPV of Model 1 were 0.756 and 0.922, of Model 2 0.775 and 0.914 and of Model 2s 0.800 and 0.908, respectively. The ROC curves of Model 1, Model 2 and Model 2s are shown in **Figures 2A-C**, respectively. The AUC values of Model 2 and Model 2s were compared using 1000 × bootstrap sampling in the validation set. The distributions of AUCs of Models 2 and 2s are shown in **Figure 2D**. There were little differences in the AUCs between Models 2 and 2s. Considering its simplicity, we recommend using Model 2s for predicting excessive ovarian responses.

### Interpretation of prediction models and development of the website-based tool

**Figure 3:**
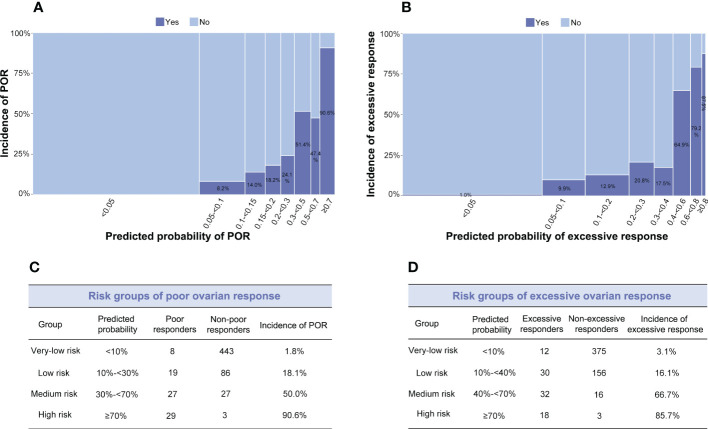
**(A, B)** Distribution between observed and predicted incidences of poor ovarian response (POR) (Model 1) and excessive ovarian response (Model 2s). The ordinate signifies the incidence, and the abscissa indicates the predicted probability: the wider the abscissa, the larger the sample size in each sub-group. **(A)** Light blue indicates the POR group; dark blue signifies the non-POR group. **(B)** Light blue indicates the excessive ovarian response group; dark blue signifies the non-excessive ovarian response group. **(C)** Risk groups of POR. **(D)** Risk groups of excessive ovarian response.

To explore the relationship between the predicted probability and the incidence of poor or excessive ovarian response, we constructed a histogram to compare them (**Figure 3**). As shown in **Figure 3A**, the predicted probability of Model 1 was classified into eight groups. The actual incidence of POR continuously increased with the increase in predicted probability. When the predicted probability was <5%, the actual incidence of POR was zero. And when the predicted probability was ≥70%, the actual incidence of POR was over 90%. Similarly, as shown in **Figure 3B**, the actual incidence of excessive ovarian response gradually increased with the increase in predicted probability. When predicted probability was <5%, the actual incidence of excessive ovarian response was 1%. The incidence of excessive ovarian response was 86% when the predicted probability was ≥70%.

The risk groups of poor and excessive ovarian response were classified based on the incidence and predicted probability in all participants enrolled (**Figures 3C, D**). When limited to the high and the very-low risk groups, both models achieved satisfactory performances. To be practical, the algorithms of Model 1 predicting POR and Model 2s predicting excessive ovarian responses were developed into a website-based tool (http://121.43.113.123:8001/) . When the user inputs the value of required indicators and clicks ‘calculate’, the predicted probability and risk group of poor or excessive ovarian response in a certain patient are displayed.

## Discussion

The basal level of serum AMH is frequently used to predict ovarian response and guide the starting dose of rFSH for COS in assisted reproductive technology (ART) clinics. However, basal assays tend to reflect the size of the resting ovarian follicle pool (24, 25). In contrast, dynamic assays of hormonal parameters during ovarian stimulation might better reflect the number of rFSH-sensitive follicles. Thus, dynamic changes (Δ) in hormone levels could be used to guide the adjustment of the rFSH dosage during COS. Our results show that the early Δinhibin B combined with basal AMH levels can better predict ovarian response. Using these indicators, we have established models for POR (Model 1) and excessive ovarian response (Model 2s), respectively. The contribution of the early Δinhibin B is 67.7% in Model 1 and 56.4% in Model 2s, while the contribution of the basal AMH level is 16.0% in Model 1 and 25.0% in Model 2s. We have built an online website according to these models to facilitate in counseling patients on their estimated ovarian response and to adjust the dose of rFSH during COS protocols, thus reducing iatrogenic poor or excessive responses.

Predicting ovarian response is not easy clinically. Theoretically, for use in everyday ART practice, a simple approach would have an advantage over any complicated or unwieldy system. Ovarian reserve markers, including basal AMH, FSH and AFC reflect the size of primordial follicle pool. Generally, the better the ovarian reserve, the better the ovarian response. However, although ovarian reserve is related to the ovarian response to external stimuli, it is still unclear whether there is a linear relationship between ovarian response and ovarian reserve. In addition, there is heterogeneity between individuals with the same ovarian reserve, probably because of inter-individual variability such as the number of FSH receptors expressed. Therefore, the ovarian response to external gonadotropins tends to be a complex biological process. Actually, Δinhibin B reflects FSH-dependent follicular growth, thus, addition of early Δinhibin B greatly improves the performance of the model for predicting poor ovarian response, from AUC of around 0.838-0.862 (5) to 0.887-0.976. Therefore, combination of early Δinhibin B and ovarian reserve markers may better predict ovarian responses and may even have the potential to replace the ultrasound tests during ovarian stimulation.

Based on the above, we established two models for predicting poor and excessive ovarian response using both basal and dynamic biomarkers. Although age was one of the potential parameters considered for inclusion in the prediction models for both poor and excessive response, it was not included finally. Explanations may as follow (1): Age is one of the most common used indicators of ovarian reserve. As mentioned above, better ovarian reserve usually leads to better ovarian response. However, a strong inter-individual variability for ovarian reserve exists within the same chronological age group (2). In both poor and excessive response prediction models, basal AMH was included finally. There are strong correlations between age and AMH, the role of age may be replaced by basal AMH, which suggests that AMH may be a better predictor than age for ovarian reserve. The AUCs and specificities of the two models were satisfactory. However, the sensitivities of Models 1 and 2s were low at 41.2% and 50.0%, respectively. The main reason for this is that both sensitivity and specificity are calculated according to a cut-off point, which divides the outcome into binary groups, i.e., poor/non-poor response or excessive/non-excessive response. However, as shown in **Figure 3**, we further grouped the cohort into subgroups according to the distribution between predicted probability and the incidence of poor or excessive ovarian response, thereby avoiding the misclassification of poor/non-poor response or excessive/non-excessive response predicted by a probability of 50%. Thus, our models can be of better clinical significance.

The role of inhibin B levels in predicting ovarian response is unclear (26–33). Previous studies indicated that inhibin B does not have a better predictive effect than other indicators in predicting ovarian response, mainly because of its variation between individuals and fluctuations within menstrual cycles (27, 29, 32). However, dynamic levels in an ART cycle could minimize these influences. Peñarrubia et al. found that day 5 inhibin B levels were the best predictor of POR in ART cycles stimulated with gonadotropins after pituitary suppression, suggesting that the exogenous FSH-stimulated inhibin B level is better than baseline determinations (34). Li et al. reported the correlation of serum inhibin B levels on basal, day 5, the day of giving hCG and the day of oocytes retrieval, respectively, in women with diminished ovarian response (35). They found that inhibin B levels on day 5 and day-hCG had more predictive value than that on day 2 or day 3. However, they did not investigate the predictive value of Δinhibin B.

Decanter et al. used only Δinhibin B, defined as day 8 inhibin B minus day 6 inhibin B, to predict POR. They discovered that a minimal increase of 300 pg/mL is needed to rule out a POR, with the sensitivity of 70% and specificity of 94% (30). Similarly, we defined the early Δinhibin B value as serum inhibin levels of day 6 minus day 2. Compared with basal levels of AMH, FSH and inhibin B, early Δinhibin B was the most effective in predicting both excessive and poor responses. Instead of identifying a cut-off value of Δinhibin B, first, we established mathematical models combining basal AMH and Δinhibin B and other variables to predict both poor and excessive ovarian responses, with AUCs and ranges of 0.948 (0.887–0.976) in the validation data of Model 1 and 0.904 (0.836–0.945) in the validation data of Model 2s. Second, in the modeling process, we applied cross-validation to identify the ideal model, indicating more stable of our models. Third, we also applied LASSO regression to minimize the potential collinearity and over-fitting of variables. In addition, we have made the algorithms into software to make the complex algorithms available in ART practice. In addition, the Δinhibin B in our study is earlier (day 6 minus day 2) than the previous study (day 8 minus day 6). From the perspective of clinical significance, our earlier Δinhibin B measured enables the clinician to adjust gonadotropin doses better.

In addition to the main predictors (Δinhibin B and basal AMH), we also found that ΔTES and basal FSH levels contributed to predicting POR. It is reasonable that high basal FSH together with low basal AMH can better reflect poor ovarian reserve, which generally results in POR. As for the variable ΔTES, a previous study demonstrated that the serum level of TES increases slightly during COS (36), which was also confirmed here. The ΔTES was selected into the prediction model of POR and its parameter estimation is positive, which means that a high value of ΔTES would predict a high risk of POR. The increased ΔTES in POR might be due to the blocking conversion of testosterone into estradiol by reduced FSH sensitivity (37). However, the contribution of ΔTES to the POR model is relatively low (3.2%), and its predictive value needs to be validated in the future.

The stimulated E_2_ level during COS is often used to predict excessive ovarian response or OHSS to take appropriate management and to minimize potential complications (38, 39). However, in this study, we discovered that the early ΔE_2_ is not of significance compared with early Δinhibin B and basal AMH in predicting excessive ovarian response. The underlying mechanism may be that there is a strong correlation between early Δinhibin B and early ΔE_2_ (Pearson’s r=0.74 in our study, data not shown), the role of early ΔE_2_ could be replaced by early Δinhibin B, which suggests that early dynamic changes in inhibin B may be a better predictor than E_2_ for adjusting rFSH dosing during COS.

Although it needs to be validated, the biological rationale of models established in this study is that basal AMH (with or without basal FSH) and Δinhibin B (with or without ΔTES) could be complementary: the former for reflecting ovarian reserve and the latter for indicating the ovarian sensitivity in a COS treatment cycle. We aim to perform further basic research to determine the mechanism of Δinhibin B in predicting ovarian response.

The strength of this study is that we established ovarian response models based on a relatively large sample size. The original data in this study are reliable because of its prospective nature. Notably, we included all patients who underwent the most common IVF treatment without selection, thus our models can be applied to most patients.

The main limitation of this study is that we included patients in a single center and further internal and external validation of the models need to be performed. Another limitation is the unstandardized inhibin B assay kits. The variations in inhibin B values when tested by different kits would limit the applicability of our model. Standardization of inhibin B measures from different test kits might solve this problem in the future to better realize the clinical application of our models.

## Conclusions

In conclusion, the early Δinhibin B levels during COS proved very informative in predicting ovarian response. We have established sophisticated models to predict excessive and poor ovarian response using novel and traditional biomarkers. Limiting the two prediction models to the high and the very-low risk groups would achieve satisfactory performances and clinical significance. The online tools based on the models we established have good potential to facilitate ART clinicians in counseling patients on their estimated ovarian response and make better decisions on treatment adjustment or cycle cancellation. Today, faster, more sensitive, and repeatable inhibin B kits based on chemiluminescence methods are available, making it possible to use this measure as a valuable additional predictor of ovarian response during COS in the future.

## Data availability statement

The raw data supporting the conclusions of this article will be made available by the authors, without undue reservation.

## Ethics statement

Approval from the Human Reproductive Ethics Committee of Peking University Third Hospital was obtained (2015sz-017). The patients/participants provided their written informed consent to participate in this study.

## Author contributions

Conceptualization: JQ, HX; Cohort enrollment and clinical data collection: JQ, RL, HW; Methology: HX, CM, GF, YH, KA; Study organization assistance: RL; Writing original draft: CM, HX, HW; Writing review and editing: all authors; Supervision: JQ. All authors contributed to the article and approved the submitted version.
