# Supplementary material for: An online tool for predicting ovarian responses in unselected patients using dynamic inhibin B and basal antimüllerian hormone levels
Source: Front Endocrinol (Lausanne). 2023 Jan 20;14:1074347. doi: 10.3389/fendo.2023.1074347 (PMC9895413; doi:10.3389/fendo.2023.1074347)
Supplement: Supplementary file 1 [file DataSheet_1.pdf]

Supplemental Materials

Supplemental table 1. Univariate analysis and the effects of each variable in models predicting poor and excessive ovarian response.

Variables	Model 1 predicting poor ovarian response				Model 2 predicting excessive ovarian response				Model 2s predicting excessive ovarian response			
	Parameter estimation	*p* value	Main effect	Total effect	Parameter estimation	*p* value	Main effect	Total effect	Parameter estimation	*p* value	Main effect	Total effect
Log[Δinhibin B]	-1.275	< 0.0001	67.7%	77.7%	1.335	0.0001	56.6%	69.2%	1.594	< 0.0001	56.4%	75.0%
Log[basal AMH]	-0.732	0.0030	16.0%	25.0%	0.765	0.0131	22.1%	33.3%	0.986	0.0006	25.0%	43.5%
ΔTES	1.194	0.0259	3.2%	6.6%			-	-			-	-
Basal FSH	0.111	0.0938	0.9%	2.2%	-0.341	0.0074	1.8%	4.1%			-	-
ΔAND			-	-	0.048	0.2637	1.7%	3.3%			-	-
Basal AND			-	-	0.055	0.1144	1.4%	3.0%			-	-
Basal LH			-	-	0.089	0.2133	0.5%	1.2%			-	-

AMH, antimüllerian hormone; FSH, Follicle stimulating hormone; TES, testosterone; AND, androstenedione; LH, luteinizing hormone.

Supplemental table 2. Performance of models predicting poor and excessive ovarian response

Measures	Model 1 predicting poor ovarian response		Model 2 predicting excessive ovarian response		Model 2s predicting excessive ovarian response	
	Training set	Internal validation set	Training set	Internal validation set	Training set	Internal validation set
AUC (95% CI)	0.910 (0.856-0.945)	0.948 (0.887-0.976)	0.896 (0.851-0.929)	0.882 (0.788-0.938)	0.875 (0.827-0.911)	0.904 (0.836-0.945)
Prevalence (95% CI)	0.133 (0.106-0.165)	0.132 (0.084-0.201)	0.140 (0.113-0.173)	0.141 (0.091-0.211)	0.140 (0.113-0.173)	0.141 (0.091-0.211)
Sensitivity (95% CI)	0.456 (0.343-0.573)	0.412 (0.216-0.640)	0.431 (0.323-0.546)	0.389 (0.203-0.614)	0.389 (0.285-0.504)	0.500 (0.290-0.710)
Specificity (95% CI)	0.978 (0.959-0.988)	0.973 (0.923-0.991)	0.980 (0.962-0.989)	0.973 (0.923-0.991)	0.984 (0.968-0.992)	0.927 (0.863-0.963)
PPV (95%CI)	0.756 (0.607-0.862)	0.700 (0.397-0.892)	0.775 (0.625-0.877)	0.700 (0.397-0.892)	0.800 (0.641-0.900)	0.529 (0.310-0.738)
NPV (95%CI)	0.922 (0.894-0.943)	0.916 (0.852-0.954)	0.914 (0.885-0.936)	0.907 (0.841-0.947)	0.908 (0.879-0.931)	0.919 (0.853-0.957)

AUC, area under the receiver operating characteristic curve; CI, confidence interval; PPV, positive predictive value; NPV, negative predictive value.

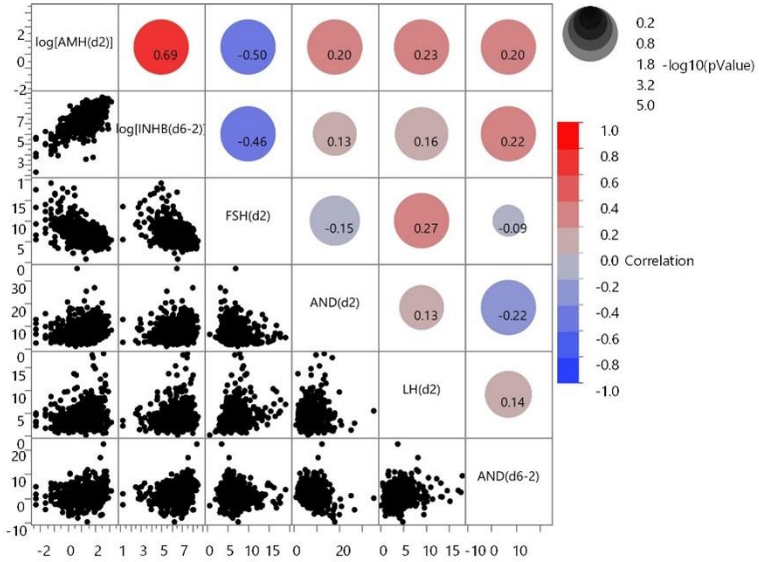

Supplemental Figure 1 The correlation between variables. AMH, antimüllerian hormone; INHB, inhibin B; FSH, Follicle stimulating hormone; AND, androstenedione; LH, luteinizing hormone.
